# Applications and Recent Advances of Low-Temperature Multicomponent Solders in Electronic Packaging: A Review

**DOI:** 10.3390/mi16030300

**Published:** 2025-03-03

**Authors:** Guodong Wu, Jingfang Shen, Ding Zhou, Muhammad Khairi Faiz, Yew Hoong Wong

**Affiliations:** 1 Department of Mechanical Engineering, Faculty of Engineering, University of Malaya, Kuala Lumpur 50603, Malaysia; wuguodong0403@gmail.com (G.W.); yhwong@um.edu.my (Y.H.W.); 2 School of Advanced Manufacturing, Guangdong University of Technology, Jieyang 522000, China; 3 Electron Microscopy Centre, Guangdong Technion-Israel Institute of Technology, Shantou 515000, China; jingfang.shen@gtiit.edu.cn

**Keywords:** low-temperature, multicomponent solder, wettability, IMC, shear strength, HEA

## Abstract

Flexible wearable devices and solar flexible units often use thermally sensitive organic materials as substrates, which are prone to thermal damage during the bonding process in 3D packaging, leading to chip deformation or failure. Multicomponent solders, with well-designed multicomponent metallic elements, exhibit unique low-melting-point characteristics. The application of low-temperature multicomponent solders in electronic packaging can significantly reduce bonding temperatures and minimize thermal damage to chips. This paper reviews the wettability and preparation methods of low-temperature multicomponent solders, and concludes the effect of different metallic elements on the solders. Additionally, this paper discusses the research on interfacial reactions, mechanical properties of low-temperature multicomponent solder joints, providing valuable insights for future research and development in this field.

## 1. Introduction

Electronic packaging is an indispensable part of the modern electronics industry [1,2]. It enables electrical [3,4], mechanical [5,6], and thermal [7,8] connections between various electronic components, ensuring they perform specific functions [9]. In the early stages, solder materials were primarily based on Sn-Pb alloys, which dominated the field of electronic packaging in the mid-to-late 20th century due to their low melting point, excellent wettability, and good electrical conductivity [10]. However, with the gradual enhancement of global environmental regulations such as the EU RoHS [11,12] and China WEEE [13,14], the development and adoption of lead-free solders have been prompted [15,16,17].

To minimize thermal damage during the packaging process, integrated circuits often adopt hierarchical packaging. Solder materials vary across different packaging levels, typically categorized into three tiers: (1) High-temperature solders (260–400 °C), such as Ag-based [18,19], copper-based [20], or Au-Sn solders [21], are used to ensure high mechanical strength and thermal stability. (2) Medium-temperature solders (180–260 °C), including Sn-Ag-Cu alloys [16] and Sn-Zn alloys [22], are applied to balance strength and process compatibility. (3) Low-temperature solders (below 180 °C), such as Sn58Bi [23], which melts at 138 °C, are used to minimize thermal damage to the previously completed packaging layers and components. Nonetheless, as modern electronic devices trend toward miniaturization and high integration [24], the demand for low-temperature solders has been increasing. Low-temperature solders [25,26] should enable soldering at reduced temperatures, minimizing thermal stress on components, protecting sensitive devices from damage, and improving production efficiency to meet the requirements of contemporary electronic packaging processes.

The selection of low-temperature solders requires careful consideration of factors such as melting point, wettability, and mechanical performance. However, despite the relatively low melting point of Sn58Bi solder, its reflow temperature still exceeds 150 °C, which poses challenges for flexible components or embedded bio-medical devices. These components often utilize organic film-based substrates, such as polydimethylsiloxane (PDMS) [27,28], polyvinyl chloride (PVC) [29], polyethylene terephthalate (PET) [27], polycarbonate (PC) [30], thermoplastic polyurethane (TPU) [31], polyethylene naphthalate (PEN) [32], and polyimide (PI) [33]. Under these conditions, Sn58Bi solder is often inadequate to meet processing requirements.

To address this issue, researchers have explored multicomponent eutectic alloys to further lower the melting point of solders. This paper reviews the preparation of multicomponent solders and the impact of various elements in low-temperature multicomponent solders (melting below 180 °C) on melting point, wettability, wetting behavior, and mechanical properties, providing valuable insights and guidance for future development and research of multicomponent solder materials.

## 2. High-Entropy Alloys and Multicomponent Solders

Multicomponent solders share many similarities in composition and properties with high-entropy alloys (HEAs). Since HEAs’ introduction in 2004, they have garnered significant attention from both academia and industry due to their innovative multi-principal element design and outstanding comprehensive properties [34,35,36,37]. This novel design significantly increases the entropy of the alloy. The remarkable properties of HEAs are closely tied to their four unique effects [38]: the high-entropy effect [39], lattice distortion effect [40,41], sluggish diffusion effect [42,43], and “cocktail” effect [44]. These effects provide ideas for the design of multicomponent solders.

However, only a few studies are available on low-temperature multicomponent solders. Among these, research on low-temperature multicomponent solders is even scarcer, just over a dozen studies [45]. This indicates that low-temperature multicomponent solders represent a novel and highly promising research direction with considerable potential for further exploration and development.

### 2.1. High-Entropy Effect of HEAs and Multicomponent Solders

The high-entropy effect refers to the enhancement of material phase stability by increasing the mixing entropy, which allows the alloy to form simple face-centered cubic (FCC) or body-centered cubic (BCC) solid solution phases, instead of complex intermetallic compounds (IMCs) or multiphase structures [46,47,48,49,50]. This single-phase solid solution structure helps to improve the material’s mechanical properties, such as strength, toughness, and durability, while also enhancing its corrosion resistance and oxidation resistance [51,52,53]. Furthermore, the high-entropy effect aids in lowering the melting point. This makes multicomponent solders particularly suitable for applications in electronic packaging and other scenarios requiring low-temperature soldering. Sezen Aksöz et al. calculated two series of multicomponent solders and compiled the results in Table 1 [54]. Their findings indicate that as the entropy ($\Delta S_{mix}$) increases, the melting point decreases, the tensile strength and microhardness increase, but the elongation decreases.

### 2.2. Lattice Distortion Effect of HEAs and Multicomponent Solders

HEAs are composed of multiple elements with varying atomic sizes, resulting in significant lattice distortion within their crystal structure. This distortion effect increases the difficulty of dislocation slip, thereby enhancing the strength and hardness of the alloy [55,56]. The lattice distortion effect also significantly hinders the growth of IMCs at the packaging interface, maintain their strength at elevated temperatures, preventing joint softening or creep [57]. Multicomponent solders possess long-term stability based on this effect.

**Figure 1:**
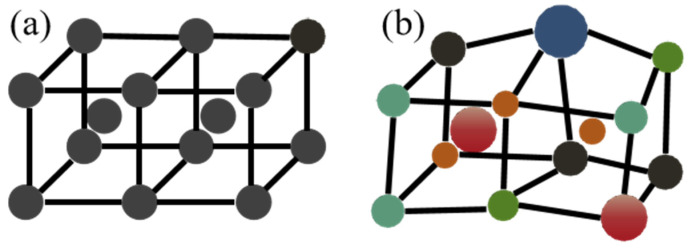
Illustrations of (**a**) a perfect BCC lattice in pure metals and (**b**) a distorted BCC lattice in multicomponent alloys. (The different colors in the diagram represent different types of elements).

**Figure 2:**
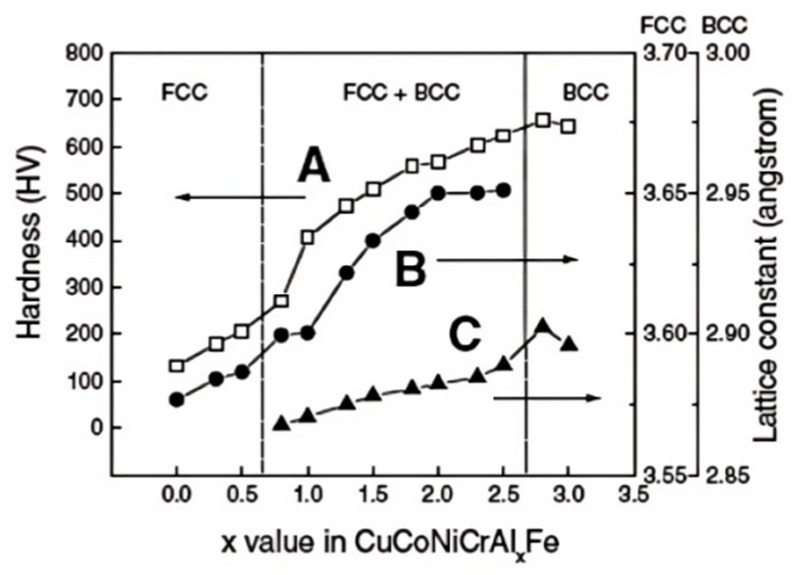
Strengthening effect of Al addition on the cast hardness of AlxCoCrCuFeNi alloys. A, B and C refer to the hardness of FCC, FCC + BCC, and BCC lattice constant, respectively.

Quanfeng He et al. have detailed the lattice distortion in HEAs, emphasizing that the distortion and associated stress primarily depend on the atomic radii and Poisson’s ratio of the constituent elements [58], as shown in Figure 1. Similarly, J. W. Yeh and his team analyzed the CuCoCrNiAlxFe multicomponent alloy system, demonstrating how increasing Al content amplified the lattice distortion effect, leading to a rise in microhardness, as illustrated in Figure 2 [59].

### 2.3. Sluggish Diffusion Effect of HEAs and Multicomponent Solders

The sluggish diffusion effect arises from the complex interactions among multiple elements in the HEAs. This effect significantly reduces atomic diffusion rates due to the intricate diffusion pathways and high activation energies [60]. Thus, multicomponent solders can maintain structural stability and have good creep resistance in high-temperature conditions. Moreover, this effect prevents excessive growth of IMCs caused by uncontrolled diffusion [61], which can significantly enhance the durability and service life of soldered joints in electronic packaging [57].

### 2.4. “Cocktail Effect” of HEAs and Multicomponent Solders

**Figure 3:**
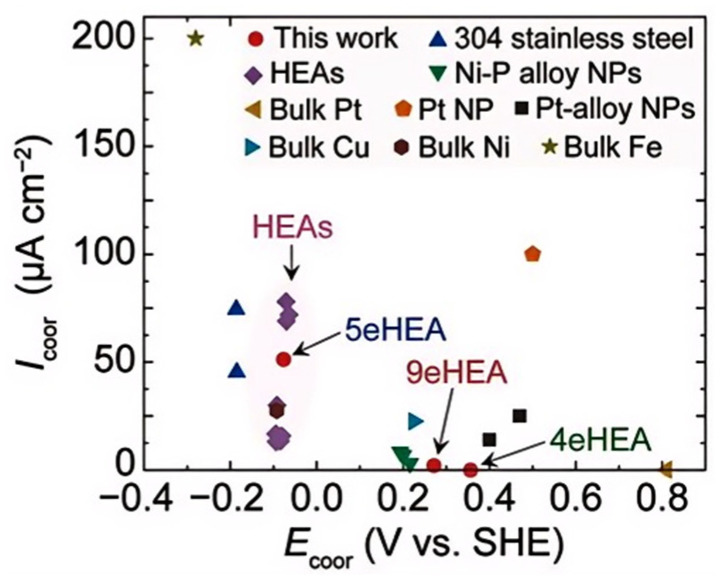
Corrosion parameters of various metals and alloys.

The “cocktail effect” is a unique phenomenon, referring to the synergistic interactions among multiple elements that result in enhanced oxidation resistance, corrosion resistance, and thermal stability, as shown in Figure 3 [62,63,64]. For example, multicomponent solders containing copper and silver elements exhibit improved wettability, while elements like chromium and nickel form dense oxide films on the surface, enhancing their oxidation resistance. Multicomponent solder achieves advantages such as a low melting point, adjustable IMC composition, and excellent mechanical properties through the synergistic interaction of its components.

### 2.5. Research Significance and Application Prospects of Low-Temperature Multicomponent Solders in Electronic Packaging

These effects above provide greater flexibility in multicomponent solder design. By adjusting the combination of elements, it is possible to simultaneously improve wettability, strength, and creep resistance while controlling the solder’s melting point and diffusion behavior. These characteristics make multicomponent solders not only promising in aerospace and energy industries but also increasingly ideal candidates for low-temperature solder in the field of electronic packaging.

## 3. Wettability of Low-Temperature Multicomponent Solders

To achieve good soldering at low temperatures, the solder must possess excellent flowability and wettability. This paper will discuss the wettability of low-temperature multicomponent solders in detail, covering its wetting behavior, measurement methods, influencing factors and current research status.

### 3.1. Interfacial Wetting Behavior

**Figure 4:**
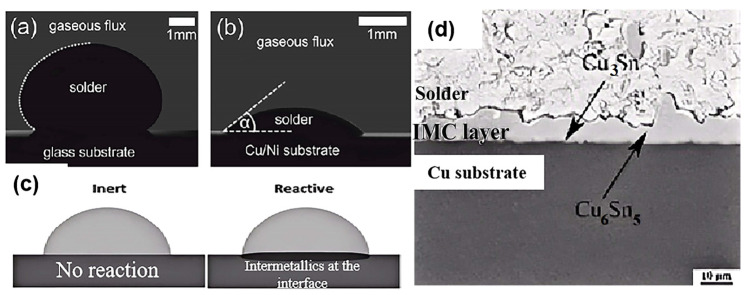
(**a**,**b**) Wetting behavior of SnBi solder on glass and Cu/Ni substrate entropy; (**c**) no-reaction interface and reaction interface; (**d**) interfacial reaction and IMC.

Flowability and wettability mean solder’s ability to contact and spread on the substrate when in a molten state. Wettability is generally assessed by measuring the contact angle (θ) formed by the molten solder on the substrate surface. The contact angle is the angle formed at the edge of the solder droplet where it meets the substrate. A smaller contact angle indicates better wettability. If the contact angle is less than 90°, the solder wets the substrate surface. However, if the contact angle exceeds 90°, the wettability is poor [65]. For example, the wettability behavior of SnBi solder on glass and Cu/Ni substrates can be observed, as shown in Figure 4a,b [66]. When the solder contacts the substrate, two types of interfaces generally exist: a non-reactive interface and a reactive interface, as shown in Figure 4c [67]. Since stable mechanical bonds are rarely formed at non-reactive interfaces, it is preferable to form an IMC at the solder–substrate interface, as shown in Figure 4d [68].

### 3.2. Wettability Analysis Method

**Figure 5:**
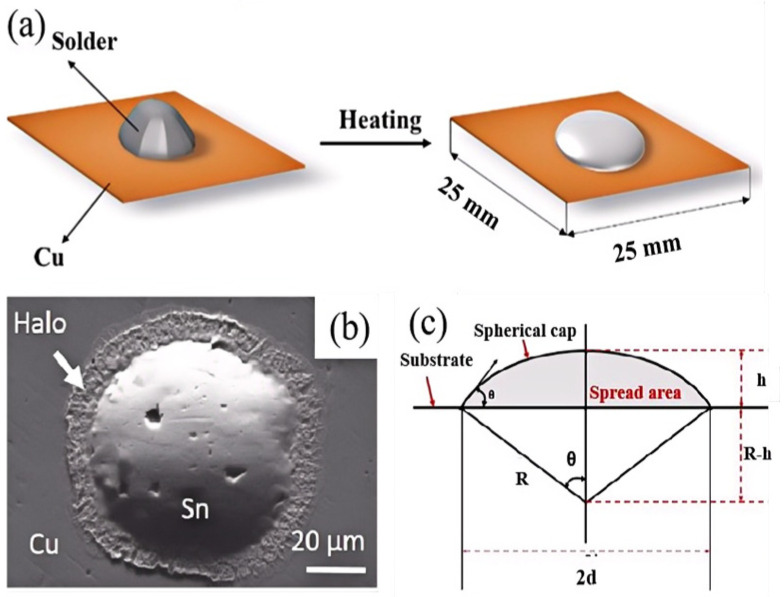
(**a**) Wetting sample preparation, (**b**) wetting spread area, (**c**) schematic diagram for the calculation of the contact angle.

In the field of soldering, a typical approach involves using approximately 0.23 g of near-spherical solder. It is placed on a smooth substrate surface, and the system is heated at a specified temperature for a set amount of time. After cooling, the solder’s wetting behavior is observed as shown in Figure 5a [69]. The wettability is generally quantified by two parameters. The first is the wetting area, which is typically measured using image analysis software, as shown in Figure 5b [70]. The second is the height and diameter of the solder joint. By measuring these, the wetting angle (θ) can be calculated using the formula $\tan(\theta )=\frac{d}{R-h}$, as shown in Figure 5c [71].

Additionally, some researchers are now using computer-based digital image simulation techniques to calculate wettability. For example, Eftal Sehirli and his colleagues analyzed the wetting angle of Sn-3Ag-0.5Cu-x(Bi, In) (x = 0.5, 1, and 2) lead-free solder alloys on a copper substrate at 300 °C, at various time intervals including the 0th, 5th, 10th, 15th, 30th, 60th, 90th, 120th, 150th, and 300th seconds. Their findings were compared to those obtained through traditional methods [72].

### 3.3. Main Factors Affecting Wettability

There are several factors that influence the wetting angle, among which the alloy composition, reflow temperature, substrate material, and surface condition of the substrate are particularly important.

We have summarized the research on the wetting angle for elemental content below 10%, and found that the effect of elemental addition on the wetting angle is not linear. For example, in the Sn-Bi-xCu alloy system, when x ranges from 0 to 0.1, the wetting angle on carbon steel decreases significantly from 39.2° to 27.8° [73]. The addition of certain elements can cause the wetting angle to initially decrease and then increase. For example, in the SnBiAg–xIn/Cu system studied by Bingwei Shen et al., the wetting angles on Cu substrate for x = 0, 0.5, 1, and 1.5 were 24.2°, 21.6°, 19.0°, and 27.0°, respectively [74].

The lattice type of the additional elements also has a significant influence on the wettability. We find that the close-packed crystal structures, such as face-centered cubic (FCC) or hexagonal close-packed (HCP), have a more significant improvement in the wettability due to their atomic arrangement and interaction with the substrate.

The reflow temperature significantly affects the wetting behavior of solders. Generally, higher temperatures result in better wetting performance. Y. Liu [75] studied the wetting angle of InZnSnBi solder on copper substrates at temperatures of 100 °C, 120 °C, 140 °C, and 160 °C, observing a decrease in wetting angle from 51.6° to 38.8°. However, the increase in temperature contradicts the goal of low-temperature soldering [72,74,76,77]. Extending the reflow time can also improve the wetting effect to some extent [70].

Additionally, the substrate type has an impact on the wetting angle. Yujie Zhang [78] and colleagues investigated the spreading rate of In58Sn on three different substrates: gold plating, matte tin, and pure copper. The results showed the best spreading effect is obtained on gold plating. Furthermore, the use of flux can significantly enhance the wetting performance. H. C. Shi and A. P. Xian [79] studied the effect of isopropyl alcohol flux in combination with six organic acid co-solvents, finding that the flux promoted wetting on copper substrates only when the reflow temperature exceeded 165 °C. Siliang [80] compared the wetting performance of Rosin Mildly Activated (RMA) flux and formic acid atmospheres, revealing that RMA had a much better wetting effect. Yi-Wun Wang [81] used activated rosin flux to achieve successful wetting on copper substrates at 160 °C.

## 4. Preparation of Low-Temperature Multicomponent Solders

### 4.1. Composition Design

The composition of multicomponent solder is complex. The significant differences in atomic radii, chemical properties, and electronegativity among these elements directly influence the alloy’s melting point, surface tension, and wetting behavior. The careful selection and precise adjustment of element ratios can ensure the solder exhibits excellent properties. For instance, elements such as Ag and Ni can enhance wetting properties, whereas Ti and Cr may negatively impact overall wetting performance due to their poorer wetting characteristics.

Studies have shown that introducing low-melting-point metallic elements, such as In and Sn, can effectively reduce the melting point of the solder, thereby enhancing wetting performance in low-temperature conditions. Strengthening elements like Cu, Ni, and Ag can effectively enhance the solder’s strength and oxidation resistance. These three metals significantly improve the mechanical properties of the solder, increasing the reliability and durability of solder joints.

Introducing reinforcement phase materials such as carbon nanotubes (CNTs), graphene (GNS), and carbides (e.g., NbC) can significantly enhance the overall performance of the solder, particularly its strength and toughness, but with negative impacts on electrical conductivity [82]. These reinforcements are especially beneficial in applications subjected to high temperatures and stresses. Thus, incorporation has emerged as a new trend in multicomponent solder design. The selection and proportioning of the reinforcement phase are pivotal in the design of multicomponent solder. To provide a clearer representation of different elements, the roles, advantages, and disadvantages of various elements in low-temperature multicomponent solders are summarized in Table 2.

### 4.2. Multicomponent Solder Preparation Techniques

With the increasing application of multicomponent solders, low-temperature multicomponent solder has gained significant attention for its advantages in electronic packaging and precision industrial welding. To meet diverse application requirements, the preparation and processing techniques for low-temperature multicomponent solders have become critically important. The choice of preparation method directly influences the microstructure, physical properties, and soldering performance of the material. There are several common solder preparation techniques, such as melting, powder metallurgy, mechanical alloying, and additive manufacturing, with melting and powder metallurgy being the most widely used.

(1) Melting Technique

The melting method is one of the most traditional and widely applied techniques for alloy preparation and is extensively used in the production of multicomponent solders. By heating multiple metal elements to their molten state, thorough mixing is achieved, then the material is solidified upon cooling to form solder.

**Figure 6:**
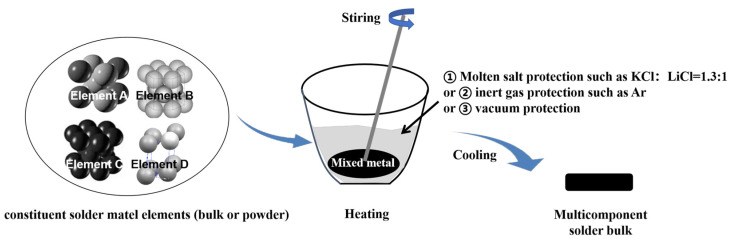
Preparation of multicomponent solder by melting method.

The basic steps of the melting process include heating, melting, stirring, cooling, and casting [152], as shown in Figure 6. First, raw materials are precisely proportioned according to the desired composition and then heated beyond their melting points in a vacuum-protected [54], inert gas (N_2_ [153] or Ar) environment, or under molten salt protection (e.g., KCl, LiCl, NdCl_3_ [154]). At high temperatures, the metal elements form a uniform liquid phase, which is stirred thoroughly to ensure homogeneity. Afterward, the liquid is cooled and cast into the desired shape. The melting technique enables the preparation of a large-volume alloy with a relatively simple process, making it suitable for industrial-scale production. Additionally, controlling the heating temperature and cooling rate allows for fine-tuning of the solder’s microstructure, such as grain size and phase composition, to optimize its physical and chemical properties [155]. However, there are limitations. High-temperature processing can cause low-melting-point elements such as In and Bi to volatilize, and lead to compositional segregation. Moreover, impurities introduced during the process can affect the solder’s purity. To mitigate these issues, the melting environment must be carefully controlled to minimize metal volatilization and oxidation, especially when preparing multicomponent solders containing low-melting-point metals.

(2) Powder Metallurgy

Powder metallurgy is another commonly used technique for preparing multicomponent solders [156,157,158]. Unlike melting, this method achieves uniform mixing of metals at relatively low temperatures, avoiding compositional segregation caused by high-temperature processing.

**Figure 7:**
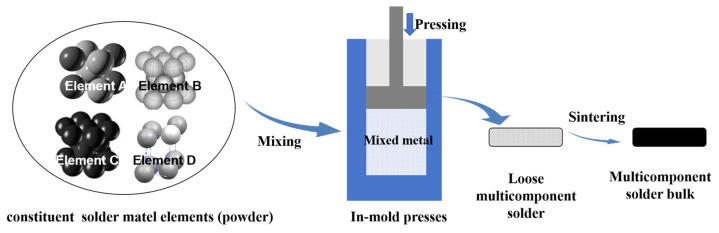
Preparation of multicomponent solder by powder metallurgy.

Powder metallurgy typically involves mixing the powdering raw materials, pressing, sintering, and post-treatment. Initially, the desired metal elements are converted into fine powders using methods like mechanical grinding or atomization [159]. These powders are then mixed in specified proportions and compacted under high pressure to form a preform. Finally, a sintering process solidifies the metal powders at relatively low temperatures to create a uniform alloy, as shown in Figure 7. Powder metallurgy offers several advantages, including reduced processing temperatures that prevent element volatilization and large-scale segregation. The method also allows precise control over the solder’s microstructure and properties by adjusting particle size and sintering conditions. For instance, using nanoscale powders can produce multicomponent solders with superior mechanical performance. Despite its benefits, powder metallurgy has limitations. The process is relatively complex, with high equipment costs, particularly during pressing and sintering. Additionally, manufacturing larger materials poses challenges, as internal porosity and structural non-uniformity may arise, potentially compromising the overall performance of the solder. By carefully selecting and optimizing these preparation techniques, multicomponent solders can be tailored to meet specific application demands, balancing their mechanical, thermal, and structural properties for advanced industrial use.

## 5. Current Research on Low-Temperature Multicomponent Solders: Interface Reactions, Microstructure Characterization, and Performance Review

The complex compositional design of multicomponent solder not only imparts exceptional performance advantages, such as high strength, excellent corrosion resistance, and superior thermal stability [160,161], but also results in more intricate phase compositions and interfacial characteristics during interactions with substrate materials [162,163]. For example, different elements in multicomponent solders may react with the substrate at varying rates, forming multiple types of IMCs. The formation and evolution of these IMCs significantly influence joint performance, including strength, toughness, fatigue resistance, and high-temperature stability.

To unravel these intricate reaction mechanisms, researchers have employed a wide range of characterization techniques, including XRD, SEM, EBSD, EDS, FIB, and TEM, to systematically analyze the phase composition of the solder, the types of IMCs, and the interfacial reaction states. Moreover, multicomponent solders exhibit complex mechanical behavior under external factors such as temperature, stress, and corrosive media.

Thus, a comprehensive understanding of multicomponent solder performance requires integrating microstructural characterization and macroscopic mechanical testing. However, current research on low-temperature multicomponent solders remains relatively limited. This paper systematically reviews existing studies on low-temperature multicomponent solders, aiming to uncover the compositional characteristics, interfacial reactions, and mechanical performance patterns of these materials.

### 5.1. Four-Element Low-Temperature Multicomponent Solders

Many low-melting-point multicomponent solders are mainly based on InSn or InSnBi matrix, and a small amount of Zn or Ga and other elements are doped. Different alloy systems will form different IMCs, such as Cu_6_Sn_5_, Cu_5_Zn_8_, CuGa, etc., which correspond to different mechanical properties. These studies provide ideas for the development of low-temperature multicomponent solders.

**Figure 8:**
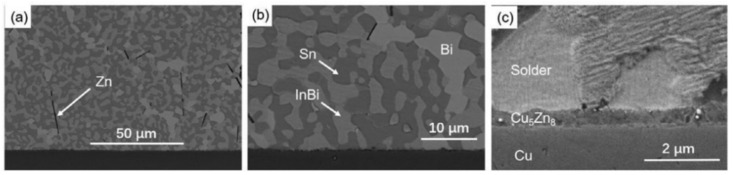
The microstructure of SnBiIn-2Zn after reflowing on Cu substrate for 10 min at 120 °C. (**a**) Needle-like Zn-rich phase, (**b**) Bi-rich phase and InBi phase in the solder matrix, (**c**) Microstructure of IMC at the interface.

**Figure 9:**
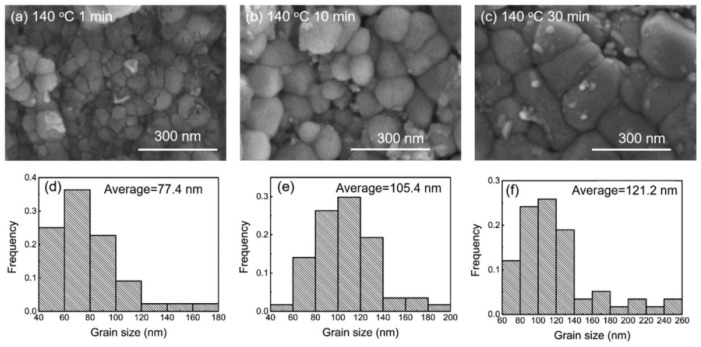
The SEM images showing the top view of IMC grains formed in the SnBiIn-2Zn/Cu interface after reflow at 140 °C for 1 min (**a**), 10 min (**b**), 30 min (**c**); (**d**–**f**) the frequency distribution of the grain size for the corresponding top view.

Yingxia Liu et al. [164] developed a 48Sn25Bi25In-2Zn solder by adding trace amounts of Zn to a SnBiIn alloy. It was prepared via vacuum induction melting at 300 °C. A small amount of the solder (0.5 mg) was placed on a Cu substrate and subjected to reflow at 120 °C, 140 °C, and 160 °C for varying durations of 5, 10, 30, 60, and 120 min to form solder/Cu joints. The microstructure of the solder consisted of large Sn phases, Bi phases, InBi phases, and elongated Zn phases, with a Cu_5_Zn_8_ IMC layer formed at the interface, as shown in Figure 8. The thickness and grain size of the IMC layer increased with rising reflow temperature and extended reflow time, as illustrated in Figure 9.

**Figure 10:**
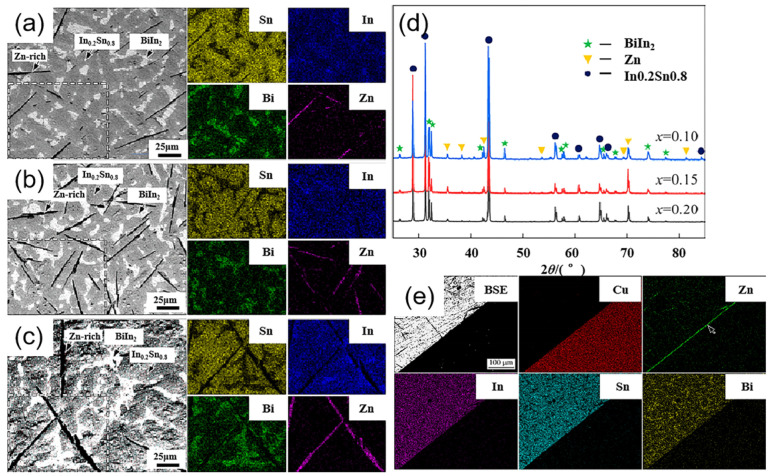
SEM-BSE images and corresponding EDS mapping of (Sn_1−x_Zn_x_)_57_(In_0.78_Bi_0.22_)_43_ solder alloy: (**a**) x = 0.10, (**b**) x = 0.15, (**c**) x = 0.20. (**d**) XRD patterns of (Sn_1−x_Zn_x_)_57_(In_0.78_Bi_0.22_)_43_ solder alloy. (**e**) SEM-BSE image and corresponding EDS mapping of (Sn_0.85_Zn_0.5_)_57_(In_0.78_Bi_0.22_)_43_/Cu.

Tian-yu Zhang et al. [165] explored low-melting-point lead-free multicomponent solders (Sn_1−x_Zn_x_)_57_(In_0.78_Bi_0.22_)_43_ (x = 0.10, 0.15, 0.20, at.%). XRD and EDS analyses revealed that the solder primarily consists of Zn-rich, BiIn_2_, and In_0_._2_Sn_8_ phases, as shown in Figure 10. The wetting behavior of the three alloy systems on a Cu plate at 160 °C was also measured. The results showed that the wetting angle decreased progressively with increasing Zn content between 0.10 and 0.20 at.%. A thin Cu_5_Zn_8_ IMC layer was formed at the interface. However, the study did not characterize the mechanical properties of the solder.

**Figure 11:**
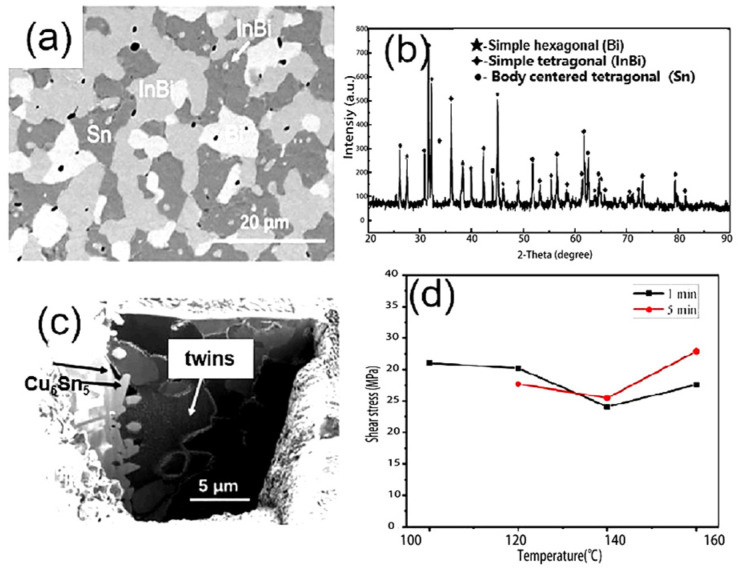
(**a**) SEM images of the original multicomponent solders. (**b**) The XRD results of the original multicomponent solders. (**c**) FIB image of the multicomponent solders reflowed at 160 °C for 5 min. (**d**) Shear stress of the multicomponent solders after reflowing on Cu substrate.

Li Pu et al. [166] prepared a low-temperature SnBiInZn solder by adding a small amount of Zn to the SnBiIn alloy. DTA analysis revealed that the alloy’s melting point was as low as 82.6 °C. The wetting angles on Cu foil were 51.6° at 100 °C and 36.6° at 120 °C. The multicomponent solders were reflowed on Cu foil at 100 °C to 160 °C for 1, 5, 10, and 20 min to form solder/Cu joints. XRD, SEM, EDS, and FIB analyses of the solder and solder joints revealed that the multicomponent solder consisted of Bi, Sn, and InBi phases, while the solder/Cu interface reaction produced Cu_6_Sn_5_ containing In and Bi atoms, as shown in Figure 11a–c. Under shear testing, the single-sided joint shear strength ranged from 19 MPa to 28 MPa. The shear strength at low (100 °C, 120 °C) and high (160 °C) temperatures outperformed that at medium temperature (140 °C), as illustrated in Figure 11d.

**Figure 12:**
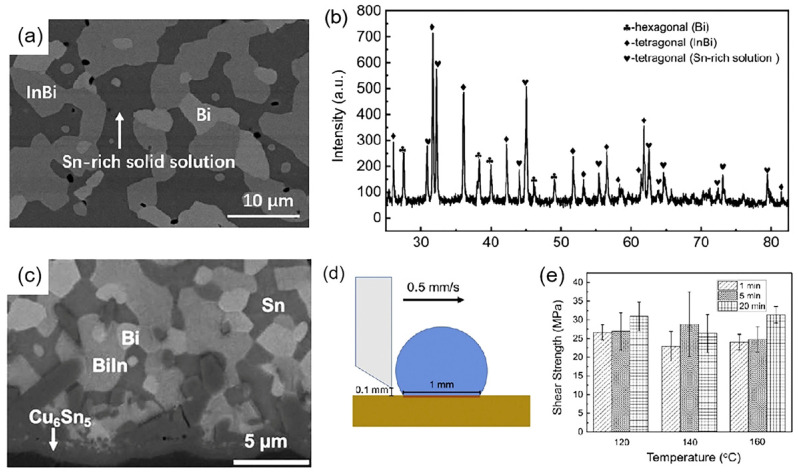
(**a**) SEM images of the original multicomponent solders. (**b**) The XRD results of the original multicomponent material. (**c**) SEM image of the multicomponent solder/Cu. (**d**) Schematic diagram showing the shear test of the solder joint. (**e**) Shear stress of the multicomponent solder after reflowing on Cu substrate.

Y. Liu et al. [75] prepared an In-Zn-Sn-Bi multicomponent solder with a melting point of approximately 80 °C using vacuum smelting. Multiple melting and solidification cycles confirmed the alloy’s stability as no significant change in its melting point was observed. XRD and SEM results revealed that the solder primarily consisted of an InBi phase, Bi phase, and Sn-rich solid solution, while the form of Zn in the alloy was not clarified, as is shown in Figure 12a,b. The solder exhibited wetting angles of 51.6°, 36.6°, 32.1°, and 38.8° on a copper substrate after reflowing at 100 °C, 120 °C, 140 °C, and 160 °C for 10 min, respectively. Solder/Cu joints prepared at these temperatures displayed an extremely thin Cu_6_Sn_5_ IMC layer at the interface, as is shown in Figure 12c. Despite the thin IMC layer, the shear strength of the single-sided joints exceeded 20 MPa, as is shown in Figure 12e.

**Figure 13:**
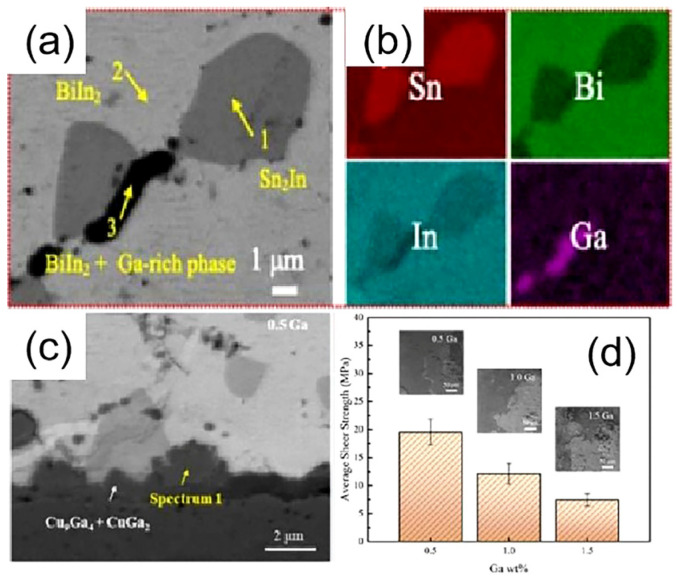
(**a**) SEM images of Sn-Bi-In-xGa solder. (**b**) The elemental mapping of Sn-Bi-In-xGa solder. (**c**) SEM image of the solder/Cu interface. (**d**) Shear stress of the multicomponent solder after reflowing on Cu substrate.

Jingyu Qiao et al. [167] incorporated trace amounts of Ga into an In-Zn-Bi alloy to produce In-Zn-Bi-xGa (x = 0.5, 1.0, 1.5 wt%) solders with melting points of 54.17 °C, 53.72 °C, and 49.84 °C, respectively. XRD, SEM, and EDS analysis revealed that the solder was composed of BiIn_2_, Sn-In, and Ga-rich phases, as shown in Figure 13a,b. After reflowing on a Cu substrate, In-Zn-Bi-xGa/Cu joints were formed, with CuGa_2_ and Cu_9_Ga_4_ IMCs observed at the interface, as shown in Figure 13c. While adding Ga effectively reduced the solder’s melting point, an increase in Ga content resulted in a significant decrease in shear strength, from 19.58 MPa to 7.45 MPa, as illustrated in Figure 13d.

**Figure 14:**
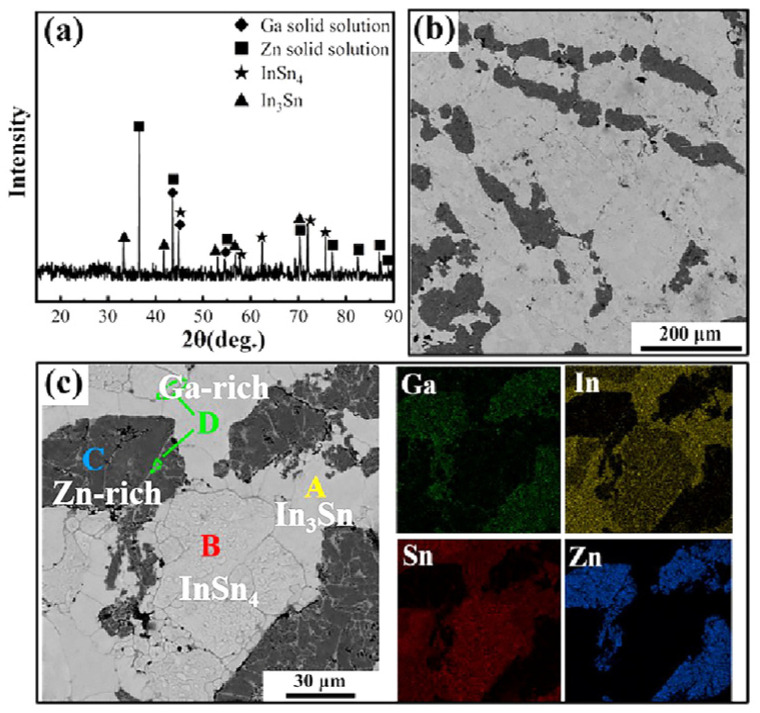
The phase constituents and microstructure of the GaInSnZn multicomponent solder. (**a**) XRD pattern. (**b**) Low-magnification BSE image and (**c**) high-magnification BSE image and corresponding elemental mappings of Ga, In, Sn and Zn elements.

Jing Ba et al. [168] synthesized a multicomponent solder with a melting point of only 9.08 °C by melting Ga, In, Sn, and Zn in equal proportions under an argon atmosphere. XRD and SEM analysis revealed that the alloy’s phase composition primarily consists of Ga solid solution, Zn solid solution, InSn_4_, and In_3_Sn, as shown in Figure 14. However, this multicomponent solder exhibits a semi-solid state at room temperature, making it unsuitable for use as a solder. Nevertheless, its extremely low melting point and unique properties make it highly suitable for use as a flexible writing conductor in flexible devices when paired with other low-temperature multicomponent solders. This also highlights Ga’s critical role in rapidly reducing the melting point of multicomponent solders.

### 5.2. Five-Element Low-Temperature Multicomponent Solders

Five-element multicomponent solders are mainly doped with Cu, Ga, Ag, Al, and other elements on the basis of SnBiInZn, respectively. Research on five-elemental multicomponent solders is still very limited, and further exploration of their performance and stability needs to be conducted.

**Figure 15:**
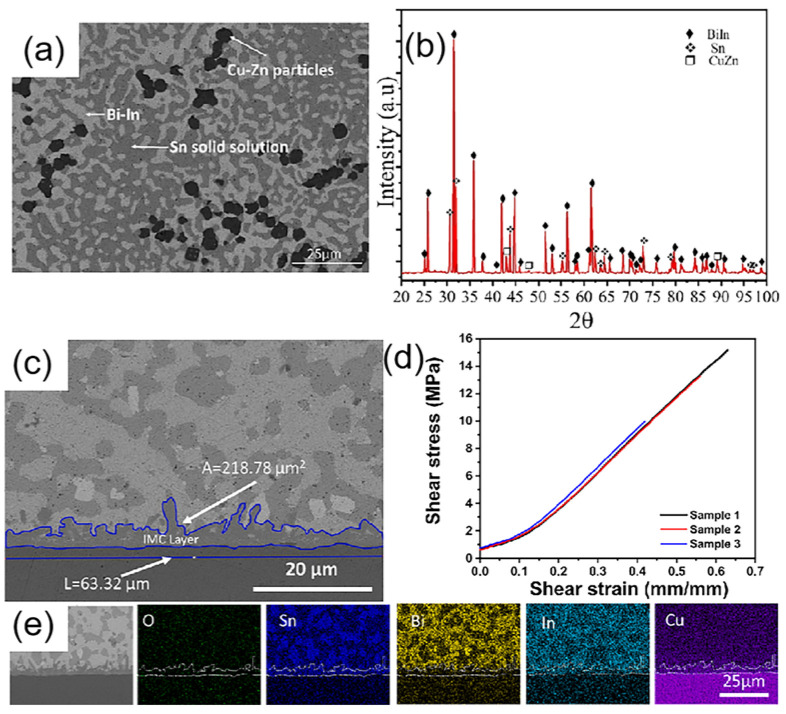
(**a**) The phase constituents and microstructure of Sn26.67Bi26.66In26.66Zn10Cu10; (**b**) XRD pattern; (**c**) BSE image of solder/Cu; (**d**) shear stress plot of the joints; (**e**) EDS mappings of the joint.

R.E. Villarreal-Loya et al. [169] prepared a Sn26.67Bi26.66In26.66Zn10Cu10 multicomponent solder using KCl-LiCl (3:1) molten salt protection during melting. DSC analysis revealed a melting point of 83.7 °C for this solder alloy. SEM and XRD analysis of the solder ingot showed that its phase composition comprises Bi-In, Sn, and Cu-Zn phases as illustrated in Figure 15. Subsequently, 0.1 mg of the solder was reflowed on a Cu substrate at 220 °C for 5 min, forming a solder/Cu single-side solder joint with a wetting angle of 51.26° and a strength of 12.94 ± 1.50 MPa. An interfacial IMC layer with an average thickness of 3.45 μm was observed. However, the specific composition of the IMC was not analyzed in this study.

**Figure 16:**
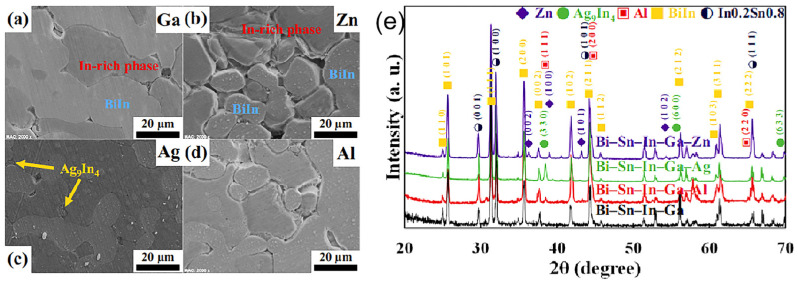
Microstructural comparison of (**a**) Bi-Sn-Ga-In, (**b**) Bi-Sn-Ga-Ag, (**c**) Bi-Sn-In-Ga-Al, and (**d**) Bi-Sn-In-Ga-Zn. (**e**) XRD analysis results of four kinds of solders.

Sang Hoon Kim et al. [170] conducted a systematic study on mixed multicomponent solders comprising Bi, Sn, In, Ga, Ag, and Al. Using a gas atomizer (Hot Gas Atomization System, PSI Ltd., Hailsham, UK), they fabricated solder micro-particles with sizes ranging from 10 to 45 μm. The specific compositions and melting points of these solders are detailed in Table 3. XRD and SEM analysis revealed diverse phase compositions for different solder formulations, as shown in Figure 16. Furthermore, the study explored the novel application of coating solder joints onto flexible PET substrates, demonstrating its potential use in flexible electronics. This research provides valuable insights into the development of multicomponent solders and their applicability in emerging electronic technologies.

**Figure 17:**
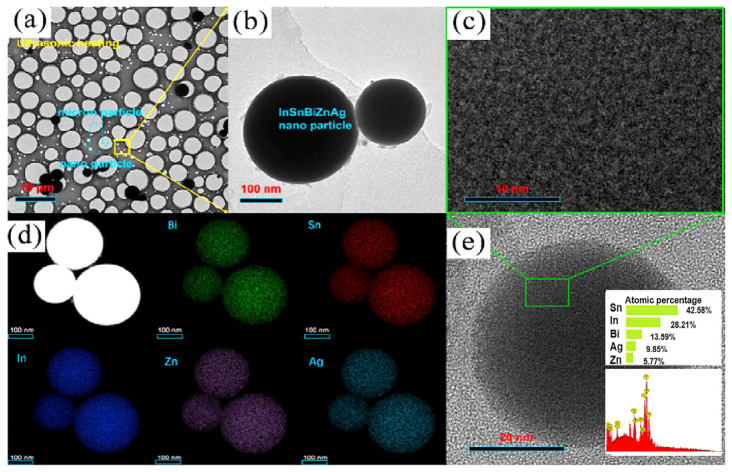
TEM images of micro- and nano-InSnBiZnAg particles. (**a**) Micro-InSnBiZnAg particles, (**b**) nano-InSnBiZnAg particles, (**c**) nanoparticles in glassy multicomponent solders, (**d**) EDS mapping of nano-InSnBiZnAg particles, (**e**) composition of nanoparticles.

Yini Chen et al. [57] successfully prepared 43In28Sn14Bi9Zn6Ag multicomponent solders as micro- and nanoparticles using melting combined with ultrasonic dispersion, as shown in Figure 17. The alloy exhibited a melting point of approximately 62.80 °C. The mixed powders were further utilized to produce solder paste, which was reflowed on a copper substrate at 115 °C for 5 min. At the solder joint interface, a Cu/Zn/Ag ternary IMC was formed. Initial tests showed a shear strength of 40 MPa for the solder joint. After aging for 0 to 15 days, the IMC thickness increased from 1.03 μm to 1.25 μm, while the shear strength slightly decreased from 40.87 MPa to 37.18 MPa. This study highlights the excellent performance and potential applications of this multicomponent alloy in low-temperature soldering, providing valuable insights for the development of high-performance low-temperature solders.

In summary, the low-temperature multicomponent solders are typically based on Sn, In, and Bi as primary alloying elements. Researchers have further explored modifying multicomponent solders’ properties by incorporating elements such as Ga, Ag, and Zn. These additional elements not only help fine-tune the melting point but also significantly influence the microstructure and interfacial reactions. For instance, even at low temperatures, these alloys can react with Cu substrates to form IMCs such as Cu_5_Zn_8_ and Cu_6_Sn_5_. Furthermore, mechanical performance tests on solder/Cu joints have shown that some solder alloys can establish strong bonding with Cu substrates even at a reflow temperature as low as 100 °C, demonstrating their promise for low-temperature applications. We compare the performance of Sn58Bi, a conventional low-temperature solder, with that of a four- and five-component multicomponent solder in Table 4.

However, current research remains insufficient, leaving several critical aspects unexplored. For example, no studies have attempted to form 3D Cu/solder/Cu joints, which hold significant potential for advanced electronic packaging. Moreover, since these solders often operate under high current densities, there is a risk of performance degradation due to heat generation, emphasizing the need for systematic analysis of their mechanical properties under working conditions. The weak points and failure mechanisms during solder joint aging have yet to be identified. Similarly, the effects of thermal shock, high-temperature aging, and electromigration on solder joint reliability demand further investigation. Addressing these gaps will provide a more comprehensive foundation for ensuring the long-term stability and reliability of multicomponent solders in practical applications.

## 6. Future Research Directions and Prospects for Low-Temperature Multicomponent Solders

The research and application of low-temperature multicomponent solders have made significant progress in recent years. However, their widespread use still faces numerous technical and production challenges. As modern industries demand higher-performance, environmentally friendly, and intelligent materials, future solder research will not only focus on improving soldering performance but also aim to drive material multifunctionality, smart design, and green manufacturing. This chapter will explore the future research directions of low-temperature multicomponent solders from various perspectives.

The unique multi-element alloy design philosophy of multicomponent solders provides great potential in the soldering field, but further optimizing alloy compositions to meet increasingly stringent industrial standards remains a key research area. Future research will continue to explore innovative element combinations, such as the incorporation of low-melting-point metals like indium and gallium with multicomponent solders, to further reduce the melting point while maintaining reliability in high-temperature and corrosive environments.

Additionally, the application of AI technology will become a crucial driving force for future multicomponent solder research. Through big data and high-throughput computation, AI can help scientists analyze and optimize alloy compositions more efficiently, predict alloy performance, and even improve soldering performance while ensuring a low melting point. This approach will accelerate the development of new materials and realize more intelligent and precise alloy designs across multiple fields.

In terms of soldering process control, future research will focus more on enhancing solder performance by adjusting the microstructure of the solder and interface. This includes controlling grain size, phase boundaries, and the thickness of IMC layers to optimize solders’ mechanical strength and extend their service life. These technological advancements will ensure more reliable performance under extreme conditions. Furthermore, as electronic devices evolve toward higher frequency, density, and power, the heat resistance, fatigue resistance, and anti-electromigration properties of solder joints will become critical research topics.

Moreover, as global green manufacturing and sustainability concepts continue to gain importance, the green manufacturing of low-temperature multicomponent solders will become a key area of future research. Reducing energy consumption during the production process, minimizing harmful emissions, and improving the recyclability of solders are all essential challenges that must be addressed.

In conclusion, the research and application of low-temperature multicomponent solders have vast prospects. In the future, with advances in alloy design, AI technology, and green manufacturing, low-temperature multicomponent solders will play an increasingly important role in electronic packaging, flexible electronics, and high-performance materials. Through innovation across multiple domains, low-temperature multicomponent solders will become a key technological enabler in future industrial and technological developments.

## Figures and Tables

**Table 1:** $\Delta S_{mix}$ and properties of two series of SnBiInZn alloy.

Series	Elements	$\Delta S_{mix}$ J/(mol·K)	Melting Point (K)	Tensile Strength (MPa)	Elongation (%)	Microhardness of Solder Matrix Phase HV (kgfmm^−2^)
1	85Sn4Bi2In9Zn	4.67	477.2	53.6 ± 0.05	22.18	20.7 ± 0.41
	75Sn14Bi2In9Zn	6.53	463.7	63.1 ± 0.06	20.73	27.10 ± 0.54
	65Sn24Bi2In9Zn	7.48	444.5	64.3 ± 0.06	18.68	27.1 ± 0.54
2	85Sn1Bi5In9Zn	4.58	476.4	48.8 ± 0.05	38.25	28.4 ± 0.57
	80Sn6Bi5In9Zn	5.93	471.8	61.2 ± 0.06	26.25	31.7 ± 0.63
	76Sn10Bi5In9Zn	6.70	469.0	63.8 ± 0.06	18.40	33.2 ± 0.66

**Table 2:** Influence of elements on the properties of solders. (All properties are at room temperature. *T*: melting point/°C; *k*: thermal conduction/W/m·K; *R*: resistivity/µΩ·cm; *β*: coefficient of thermal expansion/×10^−6^/K; *γ*: surface tension/σ (°C); *P_min_*: lowest price on 24 October 2024/USD/ton).

Elements	*T*	*k*	*R*	*β*	*γ*	*P_min_*	Specificities
Sn [83]	231.9	66	11.5	22.0	/	19,506	Sn is a cost-effective, low-melting-point metal widely used in low-temperature solder applications. It exhibits excellent compatibility with substrates like Cu and Ag, by forming stable IMCs. Due to its favorable properties, Sn is the most commonly utilized base material in low-temperature solder. Typical solder systems include Sn-Bi-based, Sn-Zn-based, and Sn-Ag-Cu-based alloys [84,85,86,87,88].
Bi [89]	271.4	8	115	13.4	300	6000	Bi and Sn can combine to form the Sn58Bi eutectic alloy, which is widely recognized for its excellent low-temperature soldering properties. The addition of Bi significantly reduces the melting point of the Sn-based solder, making it suitable for low-temperature applications. Furthermore, Bi enhances the wetting performance of the solder on Cu substrate, promoting better adhesion and improved joint reliability. This combination of properties makes Sn58Bi alloys particularly advantageous in applications requiring efficient soldering at reduced temperatures [90,91,92,93].
Zn [83]	419.5	116	5.9	30.2	510	2831	Zn exhibits a stronger reactivity with Cu and Ag substrates compared to elements like Sn. This heightened reactivity allows Zn to more readily form IMCs such as Cu_5_Zn_8_ and CuZn_2_ when interacting with Cu-based substrates. These IMCs are critical for ensuring robust metallurgical bonding at the solder joint. The pronounced reactivity of Zn with these substrates makes it an advantageous element in alloy design, particularly for applications requiring strong, durable solder joints [94,95,96].
In [97]	156.6	82	8.4	29.7	170	239,000	The addition of In effectively reduces the melting point of the alloy, making it particularly suitable for low-temperature soldering applications. However, In has relatively low mechanical strength, and when added in large amounts, it can lead to alloys with insufficient structural integrity. During the melting process, In tends to form oxides rapidly, which can adversely affect the soldering quality. Moreover, an increase In content promotes continuous growth of IMCs, potentially compromising the reliability of the solder joint. Additionally, the high cost of In presents a significant economic challenge, limiting its widespread use in commercial applications [98,99].
Ga [97]	29.8	29	27.6	18.0	/	300,000	The addition of Ga significantly reduces the melting point of multicomponent solders, making it highly advantageous for low-temperature soldering applications. Ga readily reacts with Cu substrates to form IMCs such as CuGa_2_, and Cu_9_Ga_4_. However, the inclusion of Ga also comes with drawbacks, as it tends to reduce the joint strength of the solder. This trade-off necessitates careful control of Ga content to balance the benefits of lower melting points and effective IMC formation against the potential decrease in the mechanical performance of the solder joint [100,101,102].
Cu [83]	1084.6	400	1.7	16.5		6746	Adding an appropriate amount of Cu to low-temperature multicomponent solders can effectively enhance its tensile strength and elongation. Cu acts as a reinforcing element, contributing to the formation of robust IMCs, such as Cu_6_Sn_5_, and Cu_3_Sn. These IMCs improve the mechanical integrity and bonding reliability of the solder joints. Moreover, the addition of Cu helps to refine the microstructure of the solder, distributing stress more evenly under mechanical loads, thereby improving its overall ductility and resistance to cracking. This makes Cu a crucial component in designing solder alloys for applications requiring both strength and flexibility [103,104,105].
Ag [106]	961.8	429	1.6	19.1	995	1,080,000	Ag enhances the flow and wetting capabilities of the Sn-Zn alloy solder, allowing for better bonding between the solder and the substrate, which is crucial for achieving high-quality joints. Additionally, Ag improves the mechanical properties of the alloy, such as its strength, hardness, and fatigue resistance, making it more suitable for applications requiring reliable performance under stress. However, the main downside of incorporating Ag is its high cost, which can increase the overall price of the solder alloy. Despite this, the benefits of improved wetting and mechanical performance make Ag a valuable addition to high-performance solder materials [107,108,109].
Pb [83,89]	327.5	35	20.6	28.9	/	2311	In the aerospace industry, Pb is sometimes added to solder alloys to improve both wettability and strength, which helps achieve strong, reliable bonds between the solder and substrates, particularly in complex components that require high precision. It also contributes to the alloy’s mechanical strength, enhancing the overall durability of the solder joints. However, the use of Pb in soldering materials has been heavily restricted in many industries due to environmental and health concerns, especially under regulations like RoHS. Despite these challenges, Pb remains useful in certain high-performance applications, such as aerospace, where its properties outweigh the potential drawbacks in specific scenarios. In these cases, Pb is carefully controlled and used in small quantities to balance performance and compliance with industry standards [110,111].
Ni [83]	1455	91	6.8	13.4	/	14,600	Ni is commonly added to solder alloys to refine the weld seam’s microstructure and inhibit the growth of IMC at the interface, which can lead to improved mechanical strength and a stable interface. Additionally, Ni enhances the wettability of the solder, allowing it to better spread across the substrate and form a more uniform and reliable joint. This property is particularly valuable in high-performance applications, where the strength and durability of the solder joint are crucial. By controlling the amount of Ni in the alloy, it is possible to optimize both the mechanical properties and the wettability, making it a versatile element in soldering for electronics and other advanced materials [112,113,114].
REEs: Sm, Sb [97]	1072.0; 630.6	13; 24	150; 39.5	12.7; 11.0	/	6000, 11,800	The addition of rare earth elements (REEs), such as Sm, Sb, La, Ce, and Y, to solder alloys can significantly improve both wettability and mechanical properties. These elements can lower the surface tension of the molten solder, leading to better wetting and spreading on the substrate. Additionally, REEs can refine the microstructure of the solder, contributing to improved mechanical strength, ductility, and oxidation resistance. The influence of REEs also extends to the formation of IMCs at the solder–substrate interface, where they can help control the growth of these compounds, leading to more stable and durable joints. As a result, the incorporation of REEs in low-temperature multicomponent solders is a promising approach to enhance the overall performance of solder joints, particularly in challenging applications where both high reliability and excellent wettability are required [115,116,117,118,119].
Al, Co, Mn, Mo [83,97]	/	/	/	/	/	/	Elements like Mo, Co, and Ti have a minor effect on the melting point but play a key role in forming IMCs, strengthening grain boundaries and refining the grain size. However, they can reduce wettability. While improving strength and thermal stability, their presence requires careful optimization to balance both mechanical properties and wettability for reliable solder joints [120,121,122,123,124].
IMC powder: Cu_6_Sn_5_, Sn_3_Ag [125,126,127]	/	20–40	50–100	19–21	/	/	The direct addition of IMC particles, like Cu_6_Sn_5_, refines the grain structure, improving toughness and suppressing aging IMC growth, enhancing mechanical properties, such as ductility and fracture resistance. For example, Sn_3_Ag strengthens the solder by reinforcing the matrix, forming a stable IMC with copper, and improving thermal stability. This makes the solder ideal for durable applications like electronics and aerospace [128,129,130].
BN [131,132]	/	/	10^12^	1–5	/	/	BN, known for its mechanical properties and thermal stability, reinforces the solder matrix. It enhances tensile and shear strength, improving the bond between the solder and substrate. BN also improves thermal conductivity, which benefits temperature-sensitive components in electronics and aerospace. Additionally, BN refines the microstructure, reduces defects, and prevents grain coarsening, maintaining mechanical stability, particularly under thermal stresses [133,134,135].
CNTs [136,137,138]	/	/	1–10	−1–2	/	/	CNTs significantly enhance the tensile and shear strength of low-temperature multicomponent solders. CNTs reinforce the solder by preventing excessive deformation under stress and strengthening the interface with the substrate. They also inhibit crack propagation and improve ductility, making the solder joints more resilient under dynamic conditions. Additionally, CNTs enhance the thermal and electrical conductivity of the solder, improving its performance in high-demand applications like electronics, aerospace, and automotive industries [139,140,141].
GNSs [142,143]	/	/	10^−6^	−6–0	/	/	GNSs improve tensile strength and shear strength by reinforcing the solder matrix and enhancing the interface between the solder and substrate. GNSs also prevent crack propagation and improve ductility, allowing the solder joints to withstand dynamic stresses without failure. Additionally, GNSs can enhance thermal and electrical conductivity, making them ideal for applications requiring high performance and reliability in the electronics, aerospace, and automotive industries [144,145,146,147,148].
Oxides TiO_2_, Fe_2_O_2_, SnO_2_, Al_2_O_3_, etc.	/	/	/	/	/	/	Incorporating various oxides such as TiO_2_, Fe_2_O_3_, SnO_2_, and Al_2_O_3_ into low-temperature multicomponent solders can effectively adjust the contact angle, improving wettability. These oxides also enhance the solder’s aging resistance, ensuring long-term reliability under thermal and mechanical stresses. Additionally, the inclusion of these oxides helps refine the solder’s microstructure, contributing to improved mechanical properties and stability over time [149,150,151].

**Table 3:** Solder composition and physical properties.

Component	T_onset_ (°C)	T_offset_ (°C)	∆T (°C)	Ρ (µΩ·cm)
39.3Bi28.5Sn22.6In4.6Ga5Zn	58.9	65.8	6.9	44.27 ± 0.32
39.3Bi28.5Sn22.6In4.6Ga5Ag	59.5	70.2	10.7	43.91 ± 0.30
39.3Bi28.5Sn22.6In4.6Ga5Al	59.1	67.3	8.2	46.83 ± 0.47
41.4Bi30Sn23.8In4.8Ga	61.6	69.2	7.6	43.88 ± 0.28

**Table 4:** Characteristic comparison of Sn58Bi, four-element and five-element multicomponent solder.

Solder	Melting Point	Bonding Temperature	Wettability	Shear Strength
Sn58Bi	139 °C [171]	150 °C [75]	28.1–50° [172]	Up to 68 MPa [173]
InZnSnBi [75]	83 °C	100–160 °C	35–52°	Up to 31 MPa
43In28Sn14Bi9Zn6Ag [57]	62.8 °C	85–145 °C	23–55°	/
Comparisons	Melting point of multicomponent solder is lower	Multicomponent solder can be bonded at lower temperatures	Multicomponent solders remain their wettability at low bonding temperatures	Shear strength of multicomponent solder is lower than Sn58Bi

## Data Availability

The datasets used and analyzed during the current study are available from the corresponding author upon reasonable request.

## Abbreviations

The following abbreviations are used in this manuscript:

RoHS	Restriction of Hazardous Substances
WEEE	Waste Electrical and Electronic Equipment Directive
